# Specific IgG Response against *Mycobacterium avium paratuberculosis* in Children and Adults with Crohn’s Disease

**DOI:** 10.1371/journal.pone.0062780

**Published:** 2013-05-02

**Authors:** Julien Verdier, Louis Deroche, Matthieu Allez, Caroline Loy, Franck Biet, Christelle C. Bodier, Sylvie Bay, Christelle Ganneau, Tamara Matysiak-Budnik, Jean Marc Reyrat, Martine Heyman, Nadine Cerf-Bensussan, Frank M. Ruemmele, Sandrine Ménard

**Affiliations:** 1 Institut National de la Santé et de la Recherche Médicale (INSERM), UMR989, Paris, France; 2 Université Paris Descartes, Sorbonne Paris Cité, France; 3 Department of Gastroenterology, Hôpital Saint-Louis, APHP, Paris, France; 4 UMR ISP 1282, Infectiologie et Santé Publique,INRA centre de Tours, Nouzilly, France; 5 Institut Pasteur, Unité de Chimie des Biomolécules, Département de Biologie Structurale et Chimie, Paris, France; 6 CNRS UMR 3523, Paris, France; 7 Hépato-gastroentérologie, IMAD, E.A. BIOMETADYS, Hôtel-Dieu, CHU de Nantes, France; 8 INSERM-UMR 570, unité de Pathogénie des Infections Systémiques, Groupe Avenir, Paris, France; 9 APHP, Hôpital Necker-Enfants Malades, Service de Gastroenterology, Paris, France; 10 Neuro-Gastroenterology and Nutrition Unit, Toxalim Research Center, Institut National de la Recherche Agronomique, Toulouse, France; Universita di Sassari, Italy

## Abstract

**Background and Aims:**

Presence of serum antibodies against *Mycobacterium avium paratuberculosis* (MAP) in Crohn’s Disease (CD) as a disease characteristic remains controversial. In the present work, we assessed antibody reactivity of serum and intestinal fluid against four distinct MAP-antigens, including the recently identified MAP-specific lipopentapeptide (L5P).

**Methods:**

Immunoglobulin concentrations and specificity against 3 non MAP-specific antigens: glycosyl-transferase-d (GSD), purified protein derivative from MAP (Johnin-PPD), heparin binding haemagglutinin (MAP-HBHA) and one MAP-specific antigen: synthetic L5P were determined by ELISA in gut lavage fluids from adult controls or patients with CD, and in sera of children or adult controls or patients with CD, ulcerative colitis or celiac disease.

**Results:**

Total IgA and IgG concentrations were increased in sera of children with CD but were decreased in sera of adults with CD, thereof specificity against MAP antigens was assessed by normalizing immunoglobulin concentrations between samples. In CD patients, IgG reactivity was increased against the four MAP antigens, including L5P in gut lavage fluids but it was only increased against L5P in sera. By contrast, anti-L5P IgG were not increased in patients with ulcerative colitis or celiac disease.

**Conclusions:**

A significant increase in anti-L5P IgG is observed in sera of children and adults with CD but not in patients with other intestinal inflammatory diseases. Anti-L5P antibodies may serve as serological marker for CD.

## Introduction

Crohn’s disease (CD) is a multifactorial disease that results from a combination of genetic and environmental risk factors and is associated with exacerbated immune responses against intestinal microbes [1], [2], [3]. The role of specific infectious agents in CD pathogenesis is a long lasting hypothesis. One bacterium that has raised considerable interest is *Mycobacterium avium* subspecies *paratuberculosis* (MAP) since Johne’s disease, a chronic enteritis caused by MAP in cattle, shares many clinical and histopathological similarities with CD [4], [5], [6]. As MAP has a very slow growth and is difficult to culture, its putative role in CD has previously been assessed by PCR and serological studies. Despite multiple controversial studies, recent meta-analyses point to a significant association between MAP and CD [7], [8]. MAP belongs to *Mycobacterium avium* complex (MAC) including *M. avium* subspecies *avium*, *M. avium* subspecies *hominissuis* and *M. avium* subspecies *silvaticum* that are responsible for opportunistic infections in immune-compromised individuals [9], [10], [11], [12]. One important limitation to serological studies has been the absence of a highly specific immunodominant MAP antigen. Surface-located glycopeptidolipids (GPLs), known to participate in the pathogenicity of mycobacteria, have been widely used for serological diagnosis of mycobacterial infections, although they are not strain specific [13], [14], [15]. In MAP, recent genomic and biochemical studies have shown that GPLs are substituted by a specific lipopentapeptide (L5P) [16], [17]. L5P is a major component of the outer part of the cell envelope of MAP and the target of a strong and highly specific IgM and IgG humoral response in MAP-infected animals [16], [17]. So far, L5P has not been tested in serological studies of CD patients. In this study, we have compared systemic and local IgA and IgG antibody responses of children and adults with CD to four different MAP antigens, including synthetic L5P. Due to significant differences in immunoglobulin concentrations between groups of patients, we addressed immunoglobulin specificity by normalizing the immunoglobulin concentration among samples. We observed a significant increase in IgG titers against all four antigens in gut lavage fluids (GLF) of CD patients compared to controls and a specific IgG response against L5P in sera of CD patients compared to controls or to patients with ulcerative colitis or active celiac disease.

## Materials and Methods

### Patients

In total, 203 sera were analyzed. Ninety two samples were from adults: 45 healthy controls (18–60 years old), 24 CD (mean age: 37 years old, range: 19–78), and 23 active celiac disease (mean age: 38 years old, range: 21–48). 111 samples were from children: 31 healthy controls (mean age: 10 years old, range: 5–16), 47 CD (mean age: 14 years old, range: 8–18), 33 UC (mean age: 11.5 years old, range: 2–17). Sera from adult controls were obtained from the French national blood service (EFS) and sera from pediatric controls were collected during diagnostic workup for a suspected gastro-intestinal disease which was not confirmed. Diagnosis of CD and UC was based on the ECCO and Porto IBD recommendations [18], [19], [20] (combining standard clinical, radiological, endoscopic and histological findings). Diagnosis of active celiac disease was based on the presence of anti-transglutaminase 2 IgA antibodies and on histological criteria (duodenal villous atrophy with increased numbers of intraepithelial lymphocytes) [21].

Gut lavage fluids (GLF) were also collected from 24 adult patients with CD and from 20 control volunteers. Each patient received orally 4 liters of non absorbable polyethylene glycol (PEG)-based lavage fluid (Klean-prep, Norgine). After bowel cleansing was complete, samples of the clear GLF were collected for each patient. Specimens were processed within 30 minutes of collection. Samples were centrifuged (2000 rpm, at 4°C during 5 minutes) and filtered (GF/A, Whatman Scientific Ltd). Soybean trypsin inhibitor (80 µg/ml) (Boerhinger), EDTA (15 mmol/l), phenyl methyl sulfonide fluoride (2 mmol/l), sodium azide (1 mmol/l), and bovine serum albumin were added [22], [23]. Aliquots of GLF were then stored at –70°C.

Local ethics committee approval (CPP Ile de France II, n°C08-31) was obtained for these studies and all patients or their legal guardians signed an informed consent to participate.

### Quantification of Total IgA and IgG Concentrations

Sera and GLF IgA and IgG concentrations were measured by ELISA in Immulon 2HB 96-well flat-bottomed plates (Thermo LabSystems) coated overnight at 4°C with 100 µl of 5 µg/ml mouse anti-human IgA or IgG (Abcam) in 0.1 M sodium carbonate coating buffer pH 9.6. Plates were blocked with PBS-5% fetal calf serum (FCS) before incubation with diluted sera (1∶400000 and 1∶10^6^ for IgA and IgG, respectively), diluted GLFs (1∶32000 and 1∶40 for IgA and IgG, respectively), or purified human IgA (MP Bio) or IgG (Biotrend), followed by addition of horseradish-peroxidase (HRP)-conjugated mouse anti-human IgA (Abcam) or IgG (Becton Dickinson) secondary antibodies. HRP was revealed using TMB microwell peroxidase substrate (KPL) and the reaction was stopped with 50 µl 2 N H_2_SO_4_ before reading at 450 nm using an automatic Multiskan microplate reader (Thermo LabSystems). Sera and GLF dilutions have been optimized to fit within the range of standard curve; 0.78 to 100 ng/ml for IgA and IgG.

### MAP Antigens

Purified protein derivative from MAP (Johnin-PPD) was purchased from the National Veterinary Institute (Oslo, Norway). Synthesized glycosyl transferase d (GSD) epitope 230–244 (15mer TCRRMLAFLKDKENR; mol wt, 1879 Da) was obtained from Covalab. Recombinant heparin binding haemagglutinin (MAP-HBHA) cloned from the genome of the strain MAP K-10 (ATCC BAA-968) was produced in *E. coli* and purified as previously described [24].

The lipopentapeptide L5P was synthesized manually using the standard Fmoc chemistry protocol on a 4-hydroxymethylbenzoyl resin (HMBA-AM resin, Novabiochem) as described [17].

### Immunoglobulin Specificity

Standard antigen titration assays (100 to 1 µg/ml in carbonate buffer for the 4 MAP antigens) were performed to determine the optimal coating concentrations of Johnin-PPD, GSD, L5P or MAP-HBHA. NHS pretreated plates (Covalab) were coated overnight at room temperature with 100 µl of either Johnin-PPD or GSD (20 µg/ml in 0.1 M carbonate buffer). Maxisorp microtiter plates (Nunc, Roskilde, Denmark) were coated overnight at room temperature with 100 µl of L5P (20 µg/ml in Methanol). Immulon 2HB (Thermo LabSystems, Courtaboeuf, France) were coated overnight at 4°C with 100 µl of MAP-HBHA (5 µg/ml in 0.1 M carbonate buffer). After washes with PBS-Tween and blocking in PBS-5% FCS, plates were incubated for 2 hours at 37°C with either a) 100 µl of plasma samples diluted in PBS-5% FCS to adjust IgG concentration to 500 µg/ml for GSD, or 250 µg/ml for L5P and MAP-HBHA, or 125 µg/ml for Johnin-PPD specificity assays or b) 100 µl of GLF samples diluted in PBS-5%FCS to adjust IgG concentration to 250 ng/ml for all of MAP epitopes tested. GLF IgA concentration was adjusted to 18 µg/ml for L5P and MAP-HBHA, 36 µg/ml for Johnin-PPD and 72 µg/ml for GSD. HRP-conjugated mouse anti-human IgG (1∶1000) or IgA (0.325 µg/ml) was incubated for 2 hours at 37°C and revealed as above-mentioned. Results were expressed as OD at 450 nm. Blank wells were loaded with dilution buffer i.e. PBS- 5% FCS. All results were obtained by loading the same amount of immunoglobulin except when results are presented as OD450 nm×dilution factor. In this case, OD450 nm obtained by loading the same amount of IgG are multiplied by the dilution factor required to obtain the appropriate IgG concentration. The OD450 nm multiplied by the dilution factor represents a calculated value of specificity in a same given volume of serum.

### Statistical Analysis

Statistical analysis was performed using GraphPad Prism version 5.00 (GraphPad Software, San Diego, California, USA). Results for immunoglobulin concentrations and specificity were compared among groups using the non parametric test Mann-Whitney. Results for IgA and IgG specificity were expressed as OD 450 nm and results were considered positive if above mean optical density (OD) of three blanks (threshold, indicated by a grey dashed line in figures).

## Results

### IgA and IgG Concentrations in Sera and Gut Lavage Fluids

**Figure 1:**
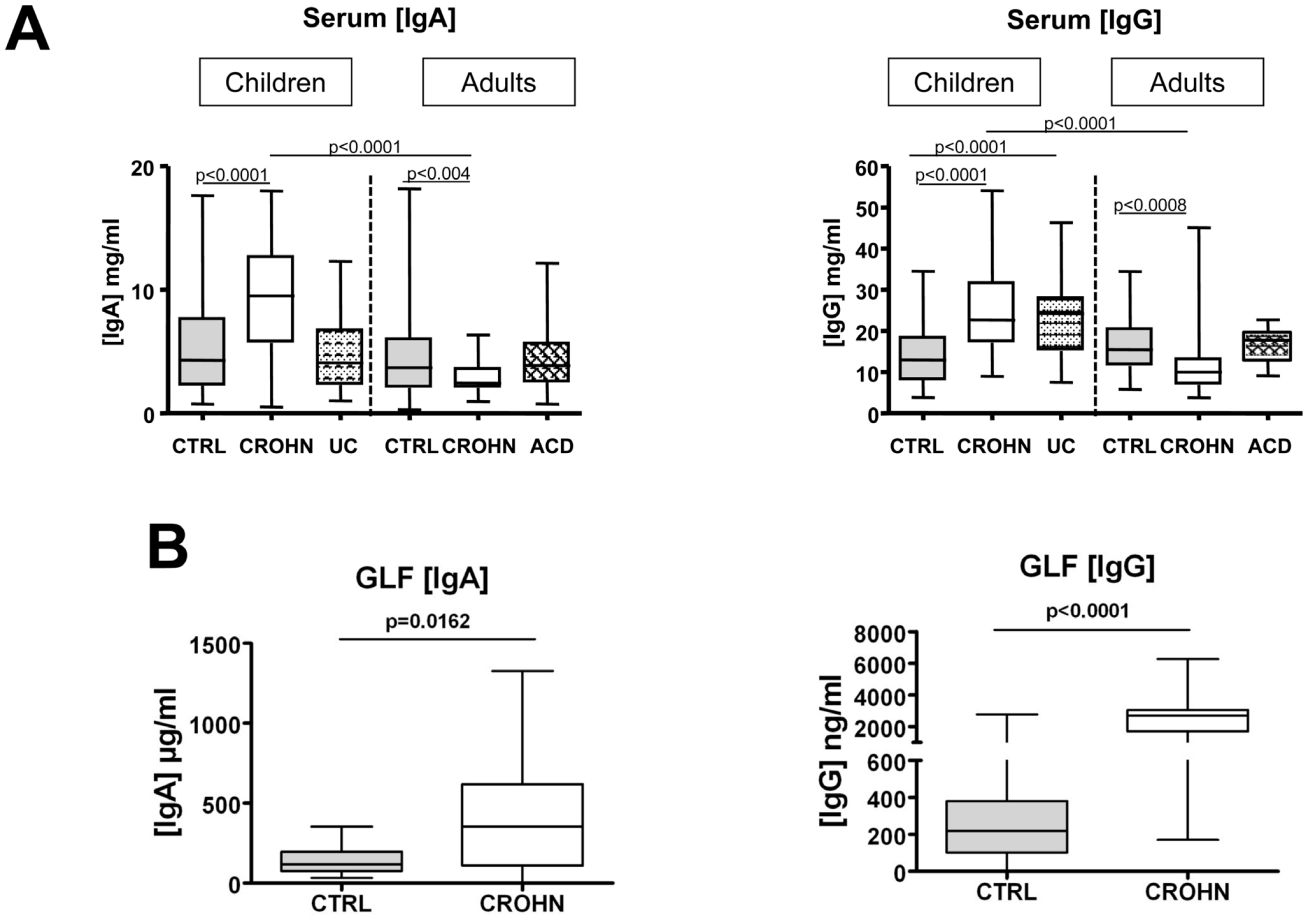
IgA and IgG concentrations in sera and gut lavage fluids. **(A)** Serum IgA and IgG concentrations measured by ELISA in children controls (n = 31), children with CD (n = 47), children with UC (n = 33), adult controls (n = 45), adults with CD (n = 24) and adults with ACD (n = 22). **(B)** Gut lavage fluids (GLF) IgA and IgG concentrations measured by ELISA in 20 adult controls and 24 adults with CD. The lower and upper lines of the boxes are the 25th and the 75th percentiles, and the lines in the boxes are the median (the 50th percentile). The whiskers extend to the highest and lowest values.

Serum IgA or IgG concentrations were not significantly different between adult and pediatric healthy controls, a result predictable as the mean age of pediatric controls was 10 years [25]. In children, IgA levels were significantly higher in CD patients than in controls whereas IgG levels were significantly higher in either CD or UC patients (Figure 1A). In adults, IgA and IgG levels were significantly lower in CD patients than in controls while no differences were observed between controls and patients with active celiac disease (ACD). As a result, serum IgA and IgG concentrations were significantly higher in children with CD than in adults with CD (3.9 and 2.3 fold increase, respectively).

Gut lavage fluids (GLF) from adult CD patients, contained higher IgA and IgG concentrations compared to GLF from controls (2.9 and 8 fold increase, respectively) (Figure 1B). This observation of marked variations in IgA and IgG concentrations between groups of patients might bias comparison of specific serological immune responses between these groups. In order to prevent any misinterpretation of results, each sample has been adjusted according to immunoglobulin concentrations before ELISA.

### Increase in Intestinal IgG Against MAP Antigens in Adult CD Patients

**Figure 2:**
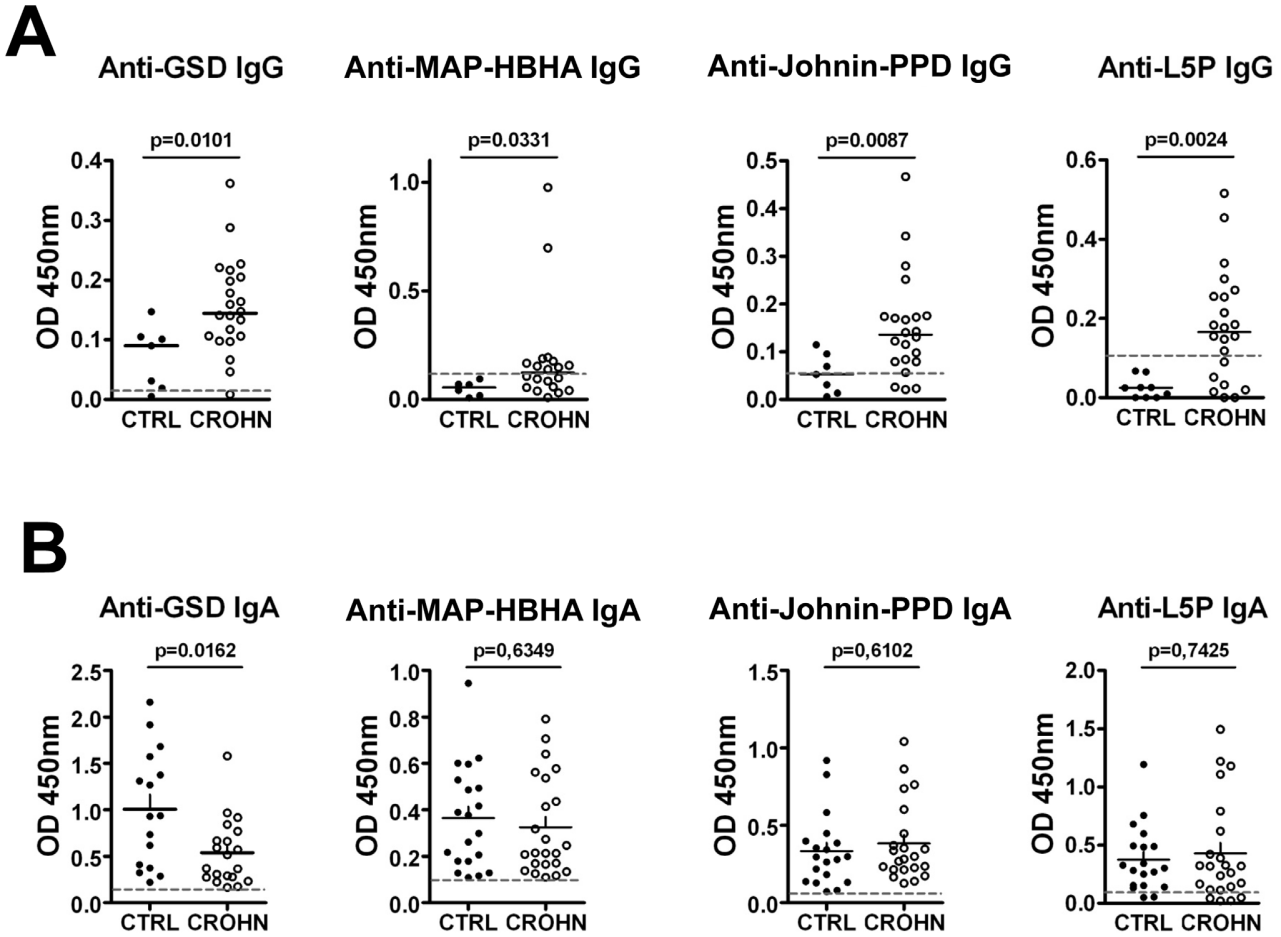
IgG and IgA specificity against MAP-antigens in GLF from adults with or without CD. **(A)** IgG specificity assessed by ELISA after normalizing IgG concentration for GSD (7 controls, 22 CD), MAP-HBHA (6 controls, 20 CD), Johnin-PPD (7 controls, 22 CD), L5P (9 controls, 22 CD). **(B)** IgA specificity assessed by ELISA after normalizing IgA concentration for GSD (16 controls, 20 CD), MAP-HBHA (20 controls, 23 CD), Johnin-PPD (19 controls, 18 CD), L5P (19 controls, 23 CD). Horizontal dashed lines indicate the threshold for specificity corresponding to 3 blanks.

IgG reactivity against all four MAP antigens was significantly higher in GLF from adult CD patients compared to controls (Figure 2A) when normalizing IgG concentration between samples. The difference between CD and controls was particularly striking for L5P as none of the controls showed any signals above threshold while substantial anti-L5P reactivity was observed in 15 out of 22 CD patients. Sensitivity and specificity for anti-L5P IgG in GFL of CD patients were respectively of 70% and 83% (Table S1A). By contrast to IgG, no difference in IgA reactivity against MAP-HBHA, Johnin-PPD or L5P was observed between controls and CD (Figure 2B). Unexpectedly, a significant decrease of anti-GSD IgA was observed in GFL of CD patients compared to controls.

### Selective Increase in Serum IgG Against L5P in Children and Adult CD Patients

**Figure 3:**
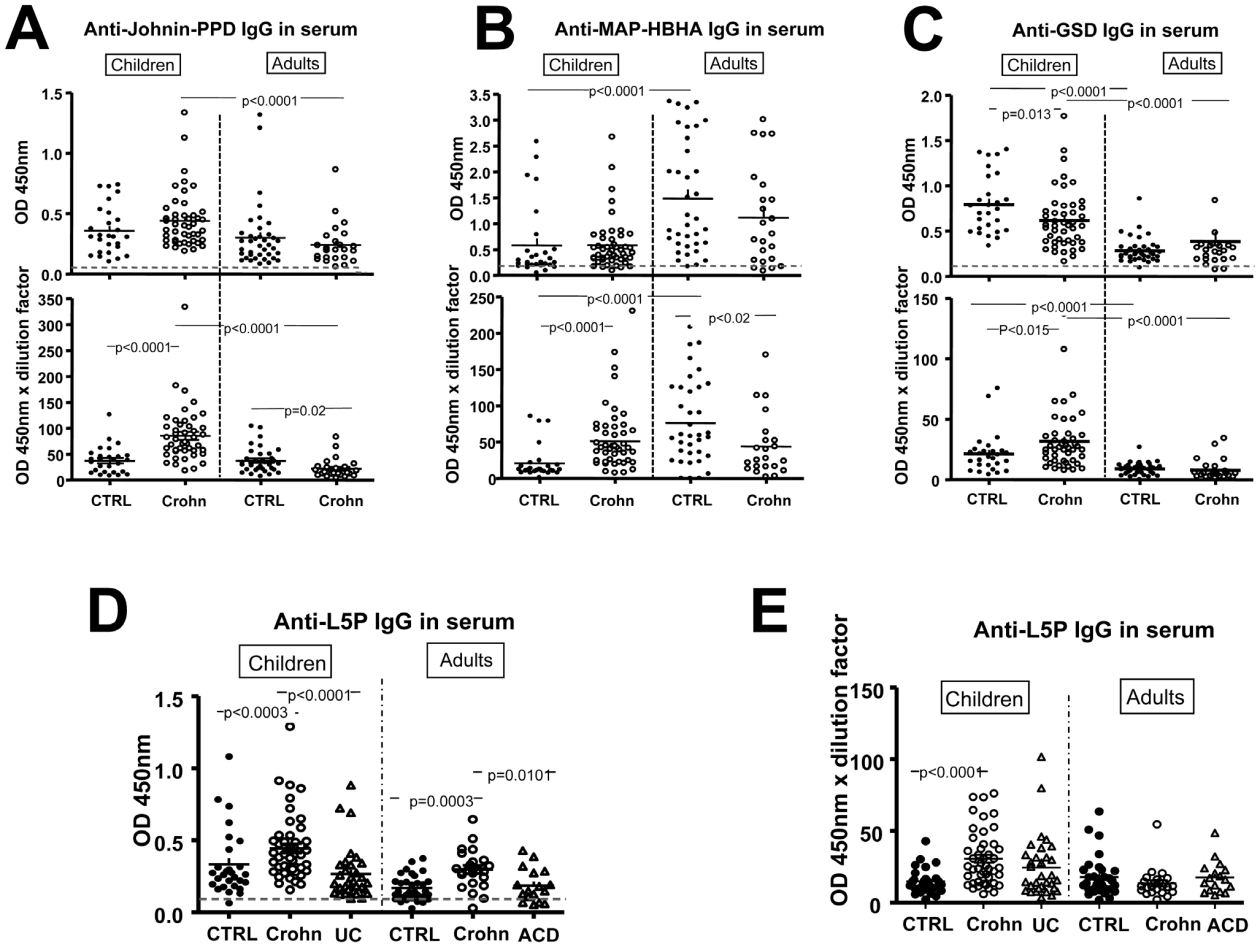
IgG specificity against MAP-antigens in sera of children and adults with or without CD, UC or ACD. IgG specificity assessed by ELISA after normalizing IgG concentration for **(A)** Johnin-PPD in children (27 controls, 46 CD) and adults (38 controls, 24 CD). **(B)** MAP-HBHA in children (28 controls, 47 CD) and adults (38 controls, 23 CD), **(C)** GSD in children (27 controls, 46 CD) and adults (31 controls, 24 CD), **(D)** L5P in children (28 controls, 46 CD, 33UC) and adults (32 controls, 23 CD, 17 ACD), **(E)** Anti-L5P IgG corrected by the dilution factor. Horizontal dashed lines indicate the threshold for specificity corresponding to 3 blanks.

When IgG concentrations were normalized, IgG titers against Johnin-PPD and MAP-HBHA were not significantly different between sera from CD patients and age-matched controls (Figure 3A–B). Unexpectedly, in children only, levels of anti-GSD IgG were significantly lower in CD patients than in controls (Figure 3C). In contrast, anti-L5P IgG titers were significantly higher in both children and adults with CD compared to respective controls (p<0.0003) (Figure 3D). Sensitivity and specificity for anti-L5P IgG are respectively 78% and 59% for adults with CD, and respectively 93% and 48% for children with CD (Table S1-C). In contrast with IgG, no significant increase in IgA levels against L5P, GSD, MAP-HBHA, and Johnin-PPD were detected between CD patients and age-matched controls (Figure S1A–D).

In order to see how variation in serosal IgG concentrations between controls and CD patients (depicted in Figure 1A) could affect the specificity test, we corrected each data by the dilution factor used to obtain normalized IgG concentrations. In children CD patients, IgG specificity against all the tested MAP antigens was then significantly increased (Figure 3A–C). In adult CD patients, specificity against Johnin-PPD and MAP-HBHA was significantly decreased (Figure 3A–B) whereas specificity against GSD and L5P was not significantly different from that of controls (Figure 3C–D). These results are consequences of higher total IgG concentrations in serum of children CD patients, and lower total IgG concentrations in serum of adult CD patients.

In CD patients, anti-L5P IgG titers did not appear to depend on disease location (Table S2) or treatment (Figure S2A–B), but the number of patients may be too small to draw definitive conclusion. However, anti-L5P IgG titers were positively correlated with disease activity based on the Harvey-Bradshaw index in children (p = 0.018), but not with disease activity based on the Crohn’s Disease Activity Index (CDAI) in adults (Figure S3A–B). In both children and adults with CD a positive correlation was also observed between serum IgG titers against L5P and serum IgG titers against Johnin-PPD, GSD or MAP-HBHA respectively (Figure S4). No correlation was observed between total IgG and anti-L5P IgG titers either in children or adults with CD, indicating that these two parameters vary independently (Figure S5A–B).

Importantly, anti-L5P IgG titers were also significantly higher in CD patients than in UC in children (p<0.0001) or ACD in adults (p = 0.0101) (Figure 3D). Sensitivity and specificity of anti-L5P IgG are respectively 78% and 65% for CD adults compared to ACD, and respectively 67% and 36% for CD children compared to UC (Table S1B–C). When anti-L5P IgG titers were corrected with dilution factor, the difference remained only significant between children with CD and pediatric controls (Figure 3E).

## Discussion

Taking advantage of the recent description of L5P as a specific MAP antigen that induces strong humoral responses [17], we compared antibody responses to L5P and to three other MAP antigens between Crohn’s disease patients and healthy controls or UC and ACD patients. Antigen specificity is usually measured in the same given volume of fluids (i.e. sera or GLF). However the diversity of immunoglobulin concentrations in GLF and serum might jeopardize the use of a same serum dilution for each sample. The humoral response would be either underestimated in adults with CD, who have fewer antibodies per volume of fluid compared to controls, or overestimated in children with CD, who have more antibodies per volume of fluid compared to control. In this study, the anti-MAP response has been analyzed by normalizing serum immunoglobulin concentrations. IgG responses against all four antigens, were significantly increased in GLF of CD patients, while only anti-L5P IgG were increased in their sera. Since no increased reactivity of plasma IgG against L5P was observed in patients with UC or ACD, using this methodology, anti-L5P IgG may be an interesting serological marker for CD.

Abnormal immune reactivity against microbiota-derived antigens is a characteristic of Crohn’s disease, attested by the detection of serum antibodies against a spectrum of microorganisms. In CD, MAP is one target of the humoral response that raised a lot of interest. A recent meta-analysis concluded to a specific association between the presence of serum anti-MAP antibodies and CD despite wide differences between published studies [7]. The latter differences might result from the use of MAP-sonicates or MAP antigens lacking specificity. To address this issue, we have compared serum reactivity of CD patients against three non-specific MAP antigens and one antigen that has been recently shown to be MAP-specific. The first antigen was Johnin-PPD, a mixture of MAP soluble proteins that has been used for diagnosis of paratuberculosis but showed cross-reactivity with PPD from other mycobacteria [26], [27], [28]. Two other antigens were MAP adhesin HBHA [29] and glycosyl transferase GSD but auto-reactivity of anti-GSD antibodies with intestinal glutathione peroxidase has been suggested [30] as well as cross-reactivity with other mycobacteria [31], [32]. The fourth antigen is a lipopentapeptide (L5P) highly specific for MAP as L5P is only recognized by serum antibodies from animals infected by MAP but not from animals infected by others mycobacteria [17]. Furthermore, in this study ELISA assay was performed with purified synthetic L5P to avoid false positive reactions obtained from native purification preparations likely to be contaminated by non specific MAP cell wall components.

Using GLF to assess intestinal humoral immunity in humans, a 7 fold increase in total IgG but only a 2.5 fold increase in total IgA was observed in GFL from CD compared to controls. In Crohn’s disease, a characteristic of the pathological local humoral response may therefore be the induction of IgG antibodies. Consistent with this hypothesis, previous studies have demonstrated abnormal recruitment of IgG plasma cells in the *lamina propria* of CD patients [33]. Accumulation of IgG-containing cells has also been observed, in a fewer extent, in gut granulomas of cattle suffering from Johne’s disease [34], [35]. Previous work of A. Fergusson and co-workers demonstrated that IgG in GLF are sensitive marker for CD activity [23], [36]. Indeed, they have reported a significant increase in specific IgG but not IgA against other intraluminal antigens, notably *Klebsiella pneumoniae,* in gut lavages of CD [36]. In keeping with these results, we observed comparable IgA reactivity against the four antigens in controls and CD patients but significant increase in anti-MAP IgG in CD patients compared to controls. The difference in specific anti-MAP IgG between CD and controls was particularly striking for L5P with 15 of 22 adult CD patients and no adult controls displaying anti-synthetic L5P IgG reactivity above the negative threshold.

The increase in IgG antibodies against all four MAP antigens in CD GLF contrasted with the selective increase in anti-L5P IgG antibodies in CD serum. This increase suggests that increased anti-MAP IgG antibodies in gut lavages are not simply due to plasma leakage but rather result from increased local responses. Specific systemic IgG against microbiota-derived antigens have been ascribed to increased translocation of bacteria or bacteria-derived products in individuals with impaired intestinal barrier [37], [38]. Alternatively, L5P, as compared to others MAP antigens tested, may be more particularly resistant to intestinal and brush border enzymes [16] and/or, as other lipopeptides, possess strong immunogenic properties [39].

Detection of intestinal humoral responses against MAP antigens, and notably against MAP-specific L5P suggests a previous exposure to MAP but is not a direct evidence of a causal role of MAP in CD. Whether MAP can be the triggering infectious agent in a subset of patients remains debated. Recent support to this hypothesis stems from evidence that NOD2 and/or autophagy genes associated with CD participate in the elimination of mycobacteria or in cytokine responses to MAP [40], [41], [42]. Although an association between CARD15/NOD2 polymorphisms and Johne’s disease was reported in cattle [43], no association has been detected between NOD2 mutations and positive MAP serology, in human CD patients. [44]. Thus, it cannot be excluded that intestinal inflammation may promote colonization by MAP, or even that antigens derived from killed-MAP and present in dairy products may induce an IgG response in the inflammatory mucosa of CD patients. Indeed, MAP can be detected in milk of productive livestock. Yet, prevalence of live MAP or MAP-derived antigens in diary food products and human exposure to this bacterium remains a controversial issue [45].

Irrespectively of the exact role of MAP in CD pathogenesis, increased levels of anti-L5P IgG might provide an interesting diagnostic marker for CD. Serum anti-*Saccharomyces cerevisiae* antibodies (ASCA) and antibodies against other microbiota-derived antigens (OmpC, I2, CBir1) are helpful adjuncts to CD diagnosis. Their specificity has been estimated between 75 to 85% compared to control but their sensitivity is much less and their presence may vary between subsets of patients [46], [47]. Interestingly, ASCA cross react with anti-mycobacteria response [48], [49]. However, ASCA are not specific for CD disease as they can also be detected in ACD [50], [51]. Anti-L5P antibodies may thus represent a valuable specific serological marker for CD as anti-L5P IgG titers were not increased in ACD or UC. Even though, in our present assay, the sensitivity was quite high (78% in adults and 93% in children) the specificity was lower (59% in adults and 48% in children). Yet, the diagnostic value of using L5P for CD (i.e. sensitivy and specificity) might be improved. Indeed, removing the lipid moiety that is not required for L5P recognition [17] or/and coupling L5P to polymers [52], [53] may help to optimize detection of anti-L5P IgG.

In CD children, the increase of total serum IgG might be a consequence of severe systemic inflammation [54]. Accordingly, in this study we observed a positive correlation between anti-L5P IgG titers and clinical score of children (BHI), still those results would need to be confirmed by increasing the number of patients. Moreover, no significant increase of anti-L5P IgG was detected in children with UC despite elevated serum IgG, suggesting that increase of anti-L5P IgG antibodies is specific to CD. However significant differences in anti-L5P IgG titers between CD and UC children patients were only observed when IgG concentrations were normalized. In adults with CD, due to decrease in total serum IgG, a significant increase in serum anti-L5P IgG titers was only observed after adjusting serum IgG concentrations. Those results underline the value of normalizing IgG concentrations when performing an immunoglobulin specificity assay in CD patients.

In conclusion, our work provides evidence of local and systemic IgG response to MAP antigens and notably to the newly defined MAP-specific L5P in Crohn’s disease. Furthermore, our work highlights the value of measuring total immunoglobulin concentration among groups before running antigen specificity assays in order to normalize results and avoid any misinterpretation. Further work is needed to address the diagnostic value of anti-L5P antibodies relative to other serological markers and/or to delineate whether it can define a distinct subgroup of patients with CD.

## Supporting Information

Figure S1 IgA specificity against MAP-antigens in sera of children and adults with or without CD. IgA specificity assessed by ELISA after normalizing IgA concentration for (A) L5P in children (26 controls, 42 CD) and adults (31 controls, 22 CD). (B) GSD in children (27 controls, 47 CD) and adults (39 controls, 24 CD), (C) MAP-HBHA in children (28 controls, 46 CD) and adults (35 controls, 24 CD), (D) Johnin-PPD in children (29 controls, 46 CD) and adults (37 controls, 23 CD). Horizontal dashed lines indicate the threshold for specificity corresponding to 3 blanks.
(TIF)

Figure S2 IgG concentrations and IgG specificity against L5P according to treatment of CD patients in children and adults. (A) Serum IgG concentrations measured by ELISA in children and adults with CD. (B) Anti-L5P IgG specificity assessed by ELISA after normalizing IgG concentration in children and adults with CD. n represents the number of patients per treatment. Medications used for treatments were anti-inflammatory drugs (5-ASA), immunosuppressants (Azathioprine, Methotrexate or 6-mercaptopurine) or corticoids (Prednisolone or Budesonide).
(TIF)

Figure S3 Correlations between IgG responses against L5P and disease severity in CD. (A) Correlation between anti-L5P IgG responses obtained after IgG normalization and Harvey-Bradshaw Index (HBI) in children with CD. (B) Correlation between anti-L5P IgG responses obtained after IgG normalization and Crohn’s Disease Activity Index (CDAI) in adults with CD.
(TIF)

Figure S4 Correlations between IgG responses against L5P and other MAP antigens in patients with CD. Spearman’s rank correlation test for correlations between IgG responses against L5P obtained after IgG normalization and other MAP antigens in children (A–C) and adults (D–F) with CD. r : Spearman correlation coefficient.
(TIF)

Figure S5 Correlations between IgG responses against L5P and serum IgG concentrations in patients with CD. Spearman’s rank correlation test for correlations between anti-L5P IgG response obtained after IgG normalization and IgG concentration in children (A) and adults (B).
(TIF)

Table S1 Sensitivy and specificity of anti-L5P IgG in GLF of adults CD (A), serum of adults with CD (B) and serum of children with CD (C).
(TIF)

Table S2 Anti-L5P IgG response obtained after IgG normalization (OD450 nm±SEM) according to disease location.
(TIF)
