# Supplementary figures and images for: Specific IgG Response against Mycobacterium avium paratuberculosis in Children and Adults with Crohn’s Disease
Source: PLoS One. 2013 May 2;8(5):e62780. doi: 10.1371/journal.pone.0062780 (PMC3642204; doi:10.1371/journal.pone.0062780)

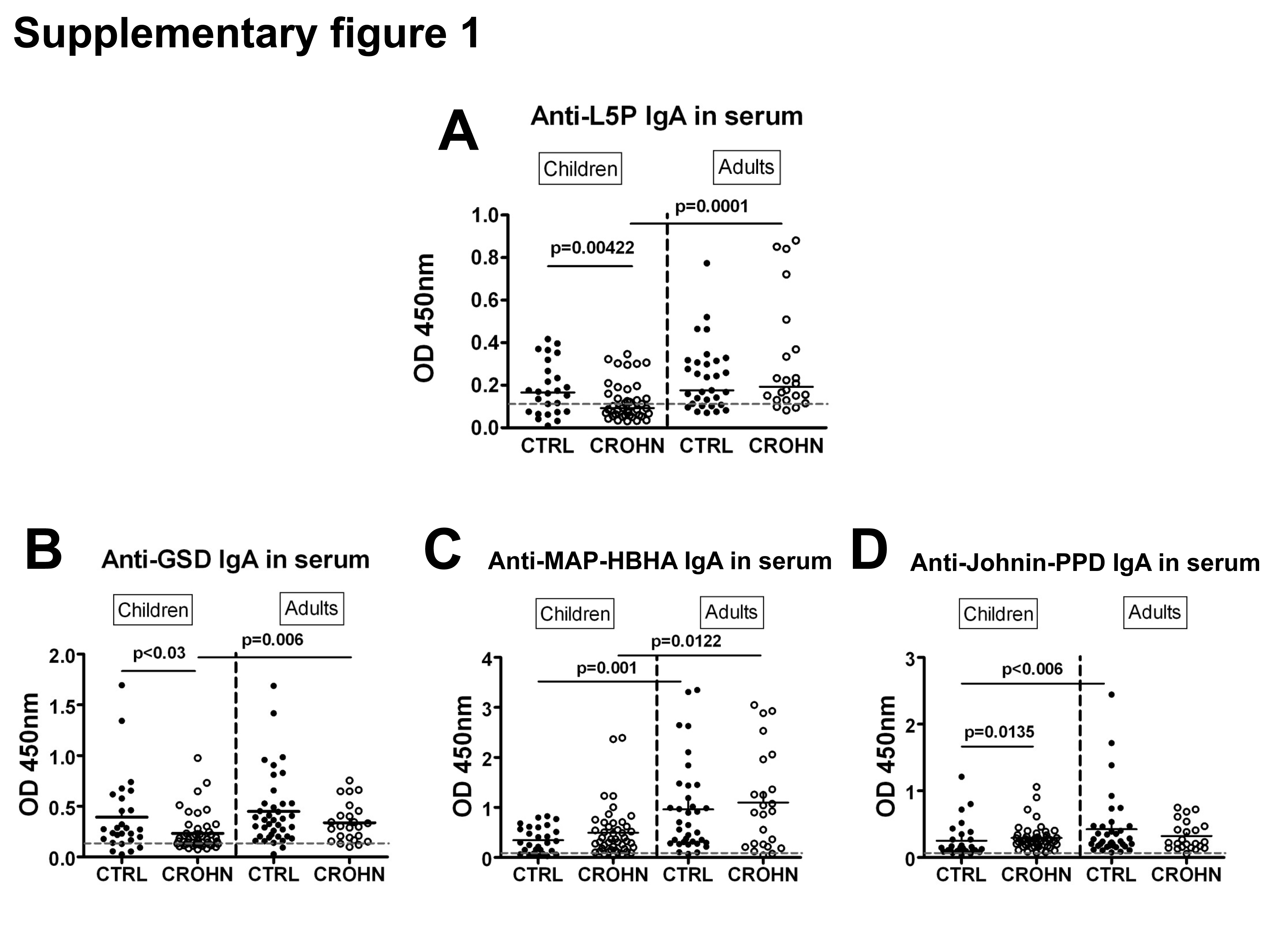

Supplement: Figure S1 — IgA specificity against MAP-antigens in sera of children and adults with or without CD. IgA specificity assessed by ELISA after normalizing IgA concentration for (A) L5P in children (26 controls, 42 CD) and adults (31 controls, 22 CD). (B) GSD in children (27 controls, 47 CD) and adults (39 controls, 24 CD), (C) MAP-HBHA in children (28 controls, 46 CD) and adults (35 controls, 24 CD), (D) Johnin-PPD in children (29 controls, 46 CD) and adults (37 controls, 23 CD). Horizontal dashed lines indicate the threshold for specificity corresponding to 3 blanks. (TIF) [file pone.0062780.s001.tif]

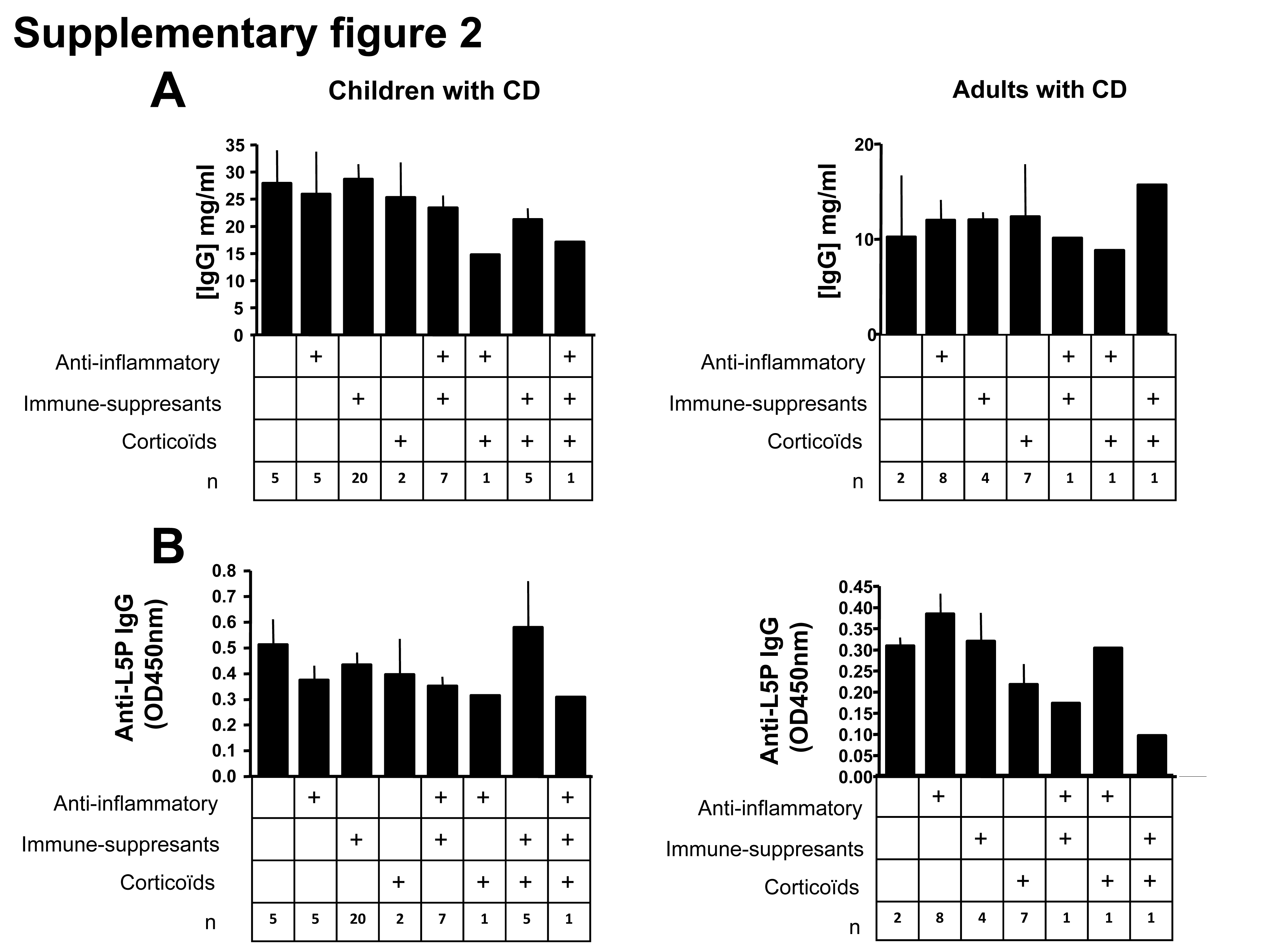

Supplement: Figure S2 — IgG concentrations and IgG specificity against L5P according to treatment of CD patients in children and adults. (A) Serum IgG concentrations measured by ELISA in children and adults with CD. (B) Anti-L5P IgG specificity assessed by ELISA after normalizing IgG concentration in children and adults with CD. n represents the number of patients per treatment. Medications used for treatments were anti-inflammatory drugs (5-ASA), immunosuppressants (Azathioprine, Methotrexate or 6-mercaptopurine) or corticoids (Prednisolone or Budesonide). (TIF) [file pone.0062780.s002.tif]

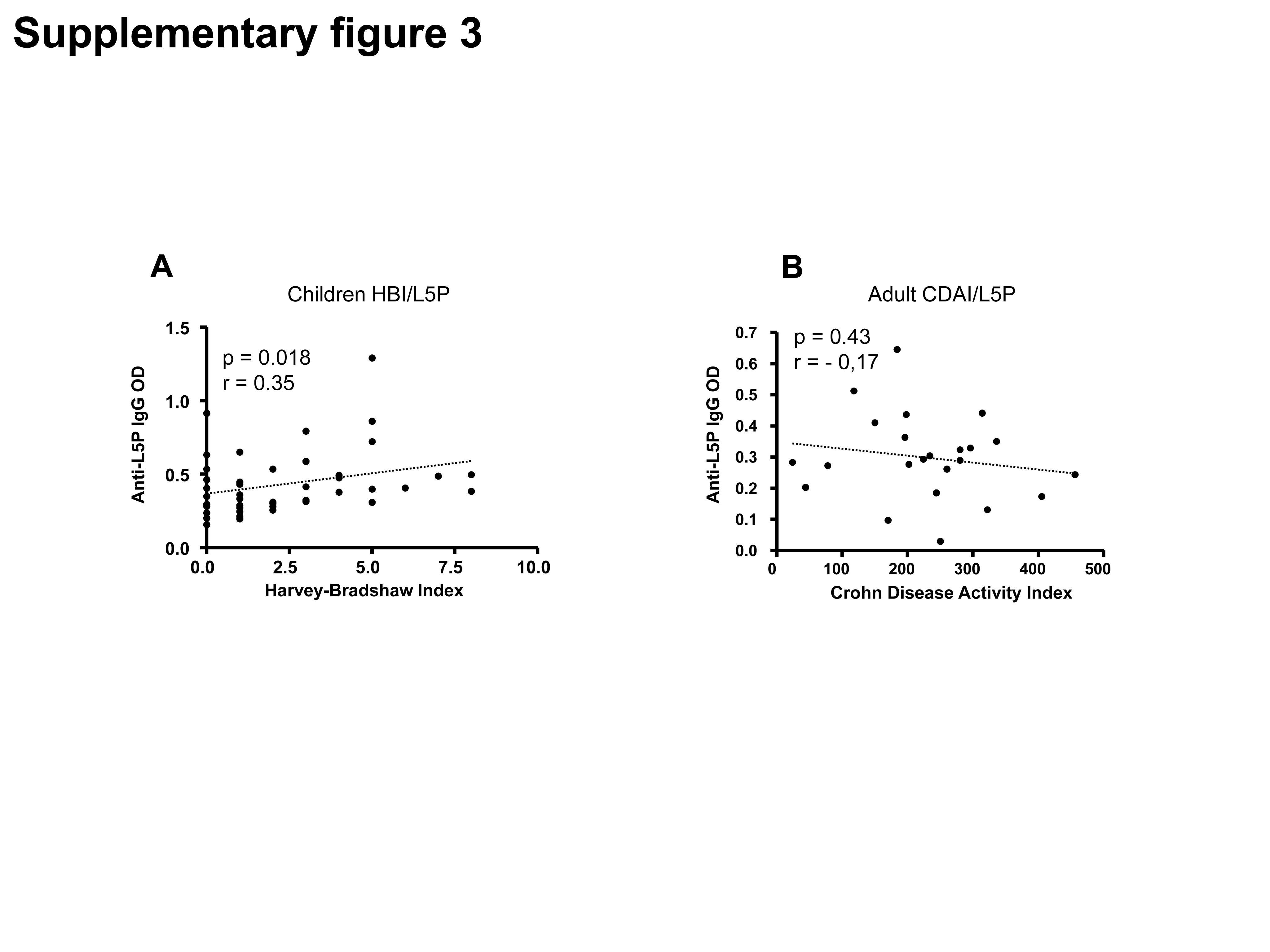

Supplement: Figure S3 — Correlations between IgG responses against L5P and disease severity in CD. (A) Correlation between anti-L5P IgG responses obtained after IgG normalization and Harvey-Bradshaw Index (HBI) in children with CD. (B) Correlation between anti-L5P IgG responses obtained after IgG normalization and Crohn’s Disease Activity Index (CDAI) in adults with CD. (TIF) [file pone.0062780.s003.tif]

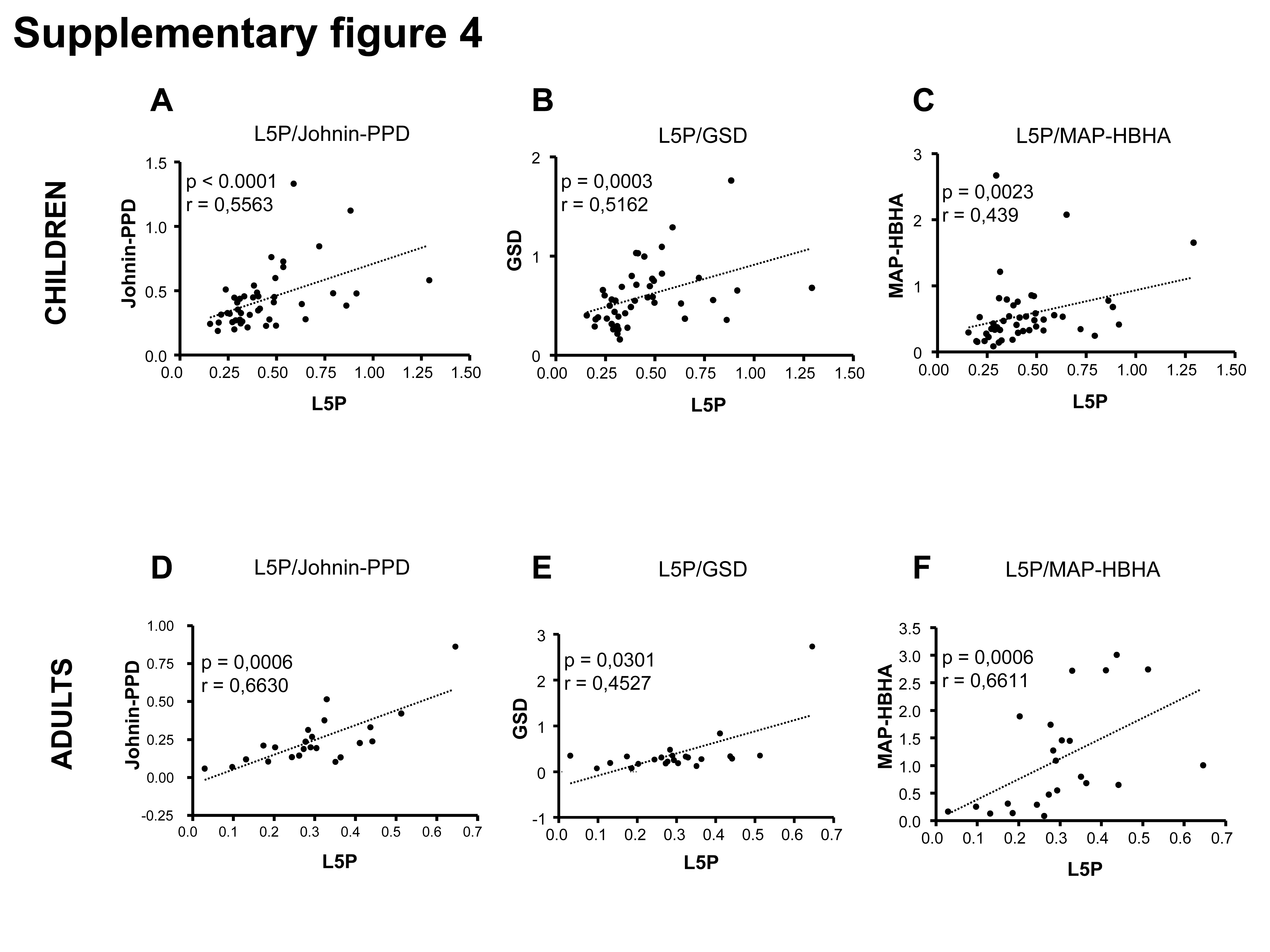

Supplement: Figure S4 — Correlations between IgG responses against L5P and other MAP antigens in patients with CD. Spearman’s rank correlation test for correlations between IgG responses against L5P obtained after IgG normalization and other MAP antigens in children (A–C) and adults (D–F) with CD. r : Spearman correlation coefficient. (TIF) [file pone.0062780.s004.tif]

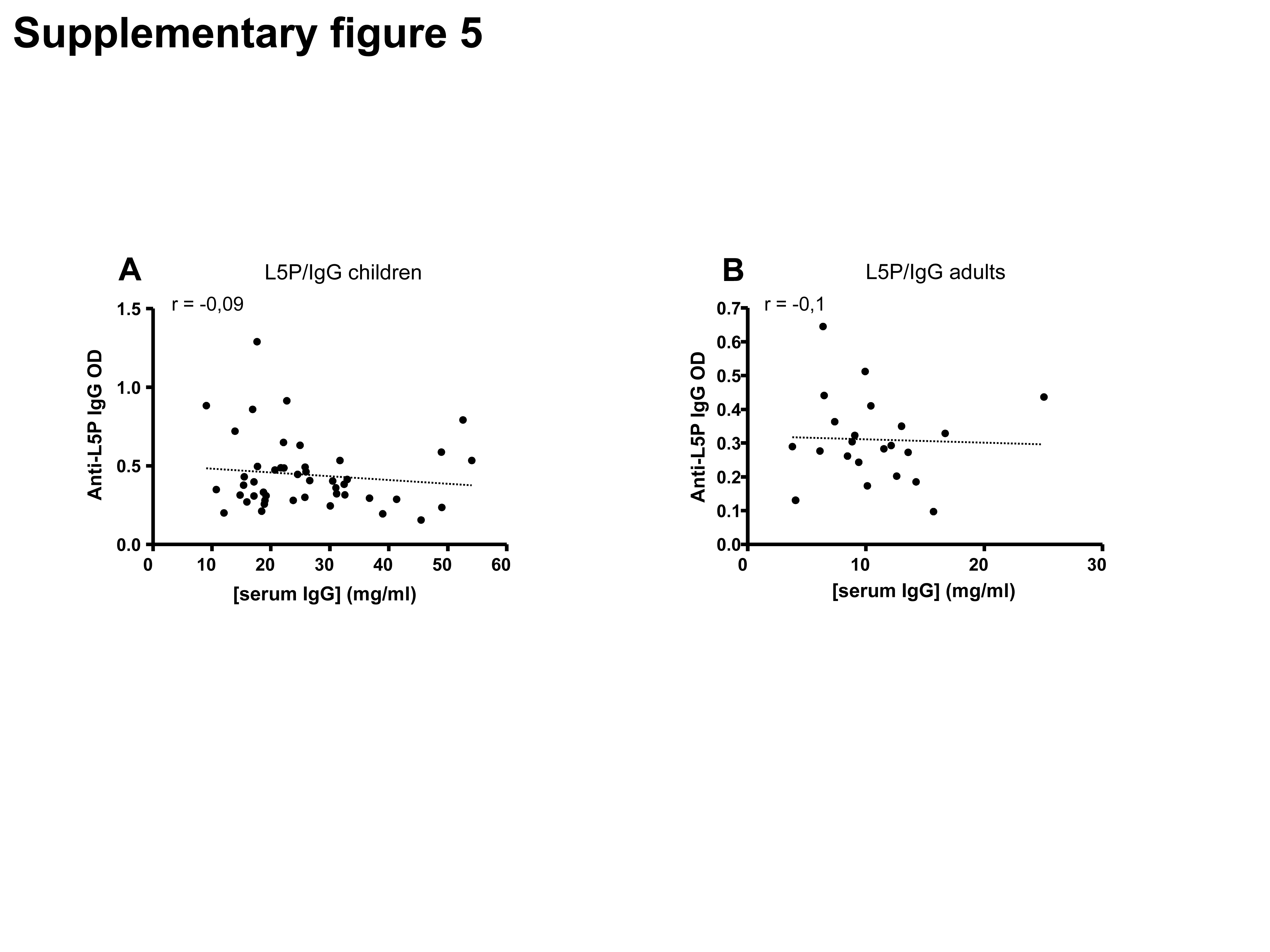

Supplement: Figure S5 — Correlations between IgG responses against L5P and serum IgG concentrations in patients with CD. Spearman’s rank correlation test for correlations between anti-L5P IgG response obtained after IgG normalization and IgG concentration in children (A) and adults (B). (TIF) [file pone.0062780.s005.tif]

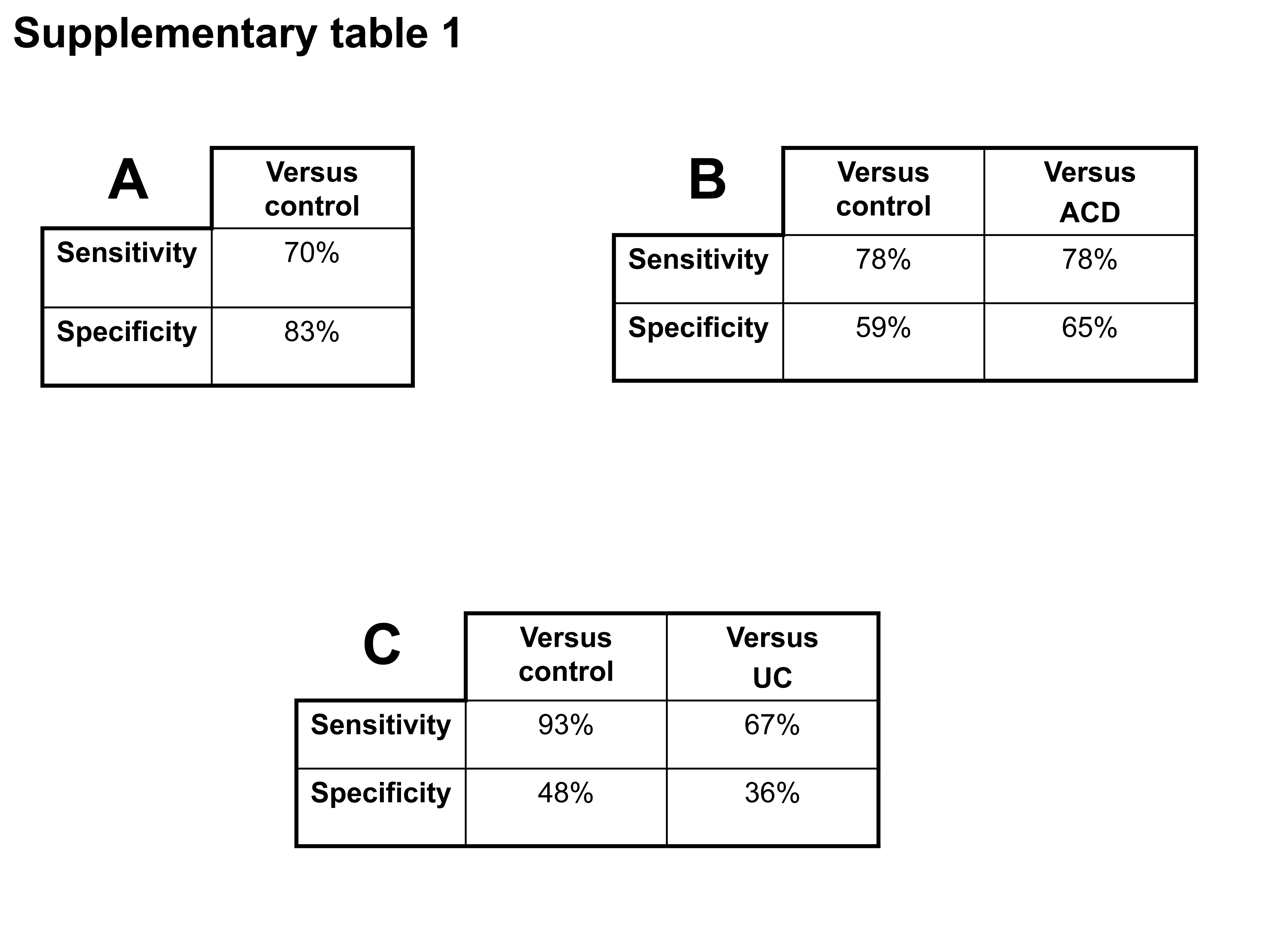

Supplement: Table S1 — Sensitivy and specificity of anti-L5P IgG in GLF of adults CD (A), serum of adults with CD (B) and serum of children with CD (C). (TIF) [file pone.0062780.s006.tif]

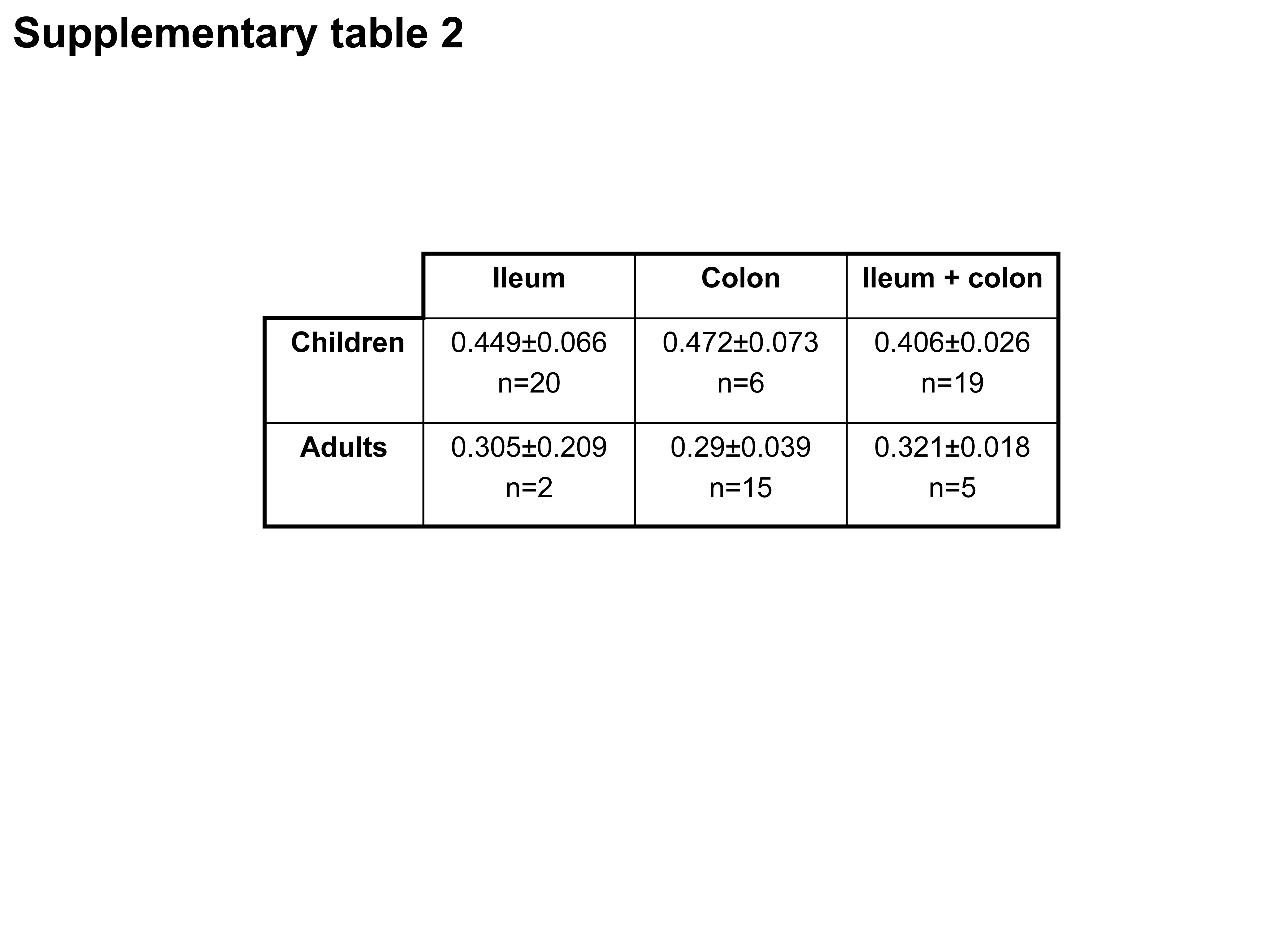

Supplement: Table S2 — Anti-L5P IgG response obtained after IgG normalization (OD450 nm±SEM) according to disease location. (TIF) [file pone.0062780.s007.tif]
